# Detorsion of Wandering Spleen Causes Significant Hypersplenism Resulting in Thrombocytopenia and Spinal Cord Injury: A Case Report

**DOI:** 10.70352/scrj.cr.25-0111

**Published:** 2025-08-14

**Authors:** Yui Innami, Yuki Mizuno, Kentaro Yamada, Kei Takasawa, Yoshifumi Ito, Kentaro Okamoto, Jun Hashimoto, Ryutaro Ohira, Konomi Shimoda, Tomoko Mizuno, Takahiro Kamiya, Toshitaka Yoshii, Masatoshi Takagi, Tomoyuki Aruga

**Affiliations:** 1 Department of Surgical Specialities, Pediatric Surgery, Institute of Science Tokyo, Tokyo, Japan; 2 Department of Orthopaedic Surgery, Institute of Science Tokyo, Tokyo, Japan; 3 Department of Pediatrics and Developmental Biology, Institute of Science Tokyo, Tokyo, Japan; 4 Department of Pediatrics, National Rehabilitation Center for Children with Disabilities, Tokyo, Japan; 5 Department of Surgical Specialities, Institute of Science Tokyo, Tokyo, Japan

**Keywords:** wandering spleen, splenic torsion, thrombocytopenia, splenectomy

## Abstract

**INTRODUCTION:**

We report the case of a child who underwent splenectomy and developed marked thrombocytopenia after detorsion due to wandering splenic torsion.

**CASE PRESENTATION:**

A 7-year-old boy who underwent laparoscopic inguinal hernia surgery developed sudden abdominal pain 2 days later. Contrast-enhanced CT revealed a poor contrast effect in the spleen, and emergency surgery was performed based on the diagnosis of splenic infarction. The spleen was not fixed to the retroperitoneum and was twisted at 720° around the splenic hilum. However, the color tone improved after detorsion of the spleen; therefore, the spleen was preserved. The next day, the patient developed spinal cord injury due to marked thrombocytopenia and epidural hematoma, and emergency hematoma removal surgery was performed. As the patient continued to depend on platelet transfusion, laparoscopic splenectomy was performed. The patient’s platelet counts rapidly increased after surgery. Since then, the patient has undergone treatment and rehabilitation for the spinal cord injury, and his neurological symptoms have improved.

**CONCLUSIONS:**

Preserving the spleen is recommended for wandering splenic torsion, especially in children. In the present case, splenectomy was necessary because of rapid thrombocytopenia caused by increased splenic function after detorsion of the spleen; however, there have been no similar case reports in the past. This condition can be potentially dangerous and can lead to serious complications.

## INTRODUCTION

A wandering spleen is rare; however, when stem torsion occurs, it causes splenic infarction, which manifests as acute abdominal pain.^1)^ If blood flow resumes after detorsion, the spleen can be preserved and the prognosis is usually good. We report the case of a child who developed thrombocytopenia and spinal cord injury due to epidural hematoma after detorsion and required splenectomy due to a prolonged dependence on blood transfusion.

## CASE PRESENTATION

A 7-year-old boy who underwent laparoscopic surgery for an inguinal hernia at our hospital developed sudden abdominal pain on the morning of the 2nd day after surgery. He was originally a very low-birth-weight baby, born at 27 weeks and 5 days of gestation, weighing 704 g. He had a history of clipping surgery for patent ductus arteriosus and bilateral tonsillectomy for obstructive sleep apnea and had been visiting our hospital regularly for short stature.

**Fig. 1:**
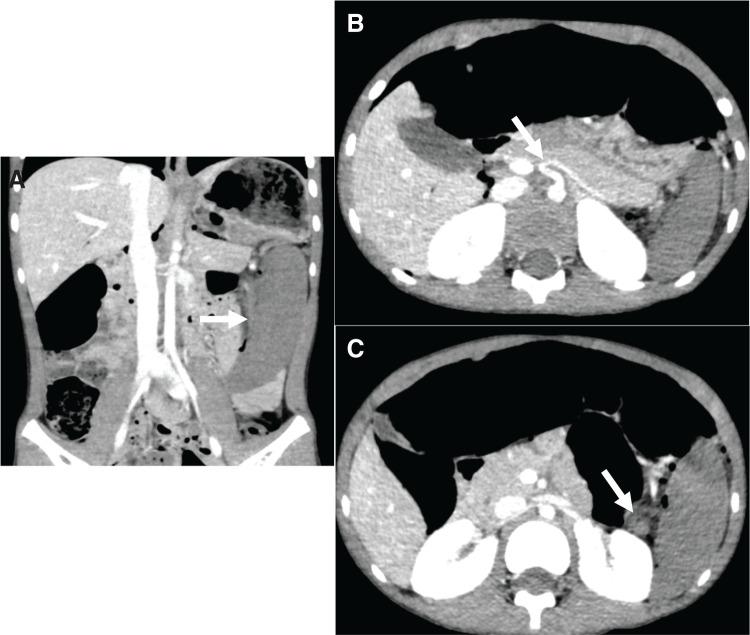
Abdominal CT scans (**A**) The coronal image reveals nonenhancement of the spleen (white arrow). (**B**) The axial image reveals the blood flow from the celiac artery to the main splenic artery (white arrow). (**C**) The axial image reveals the twisting of the blood vessels and disruption of blood flow at the splenic hilum (white arrow).

On arrival at the hospital, he had tenderness, mainly in the pericardial area; however, no peritoneal irritation symptoms such as recurrent pain or muscular defense were noted. Although blood tests showed only mild inflammatory findings, contrast-enhanced CT showed the poorly contrasted spleen (**Fig. 1A**). The blood flow from the celiac artery to the main splenic artery was observed, but vascular torsion and interruption of blood flow were observed at the splenic hilum (**Fig. 1B**, **1C**). Since abdominal ultrasonography failed to confirm splenic artery blood flow in the splenic hilum, emergency laparoscopic surgery was performed with a diagnosis of splenic infarction at the evening of onset.

**Fig. 2:**
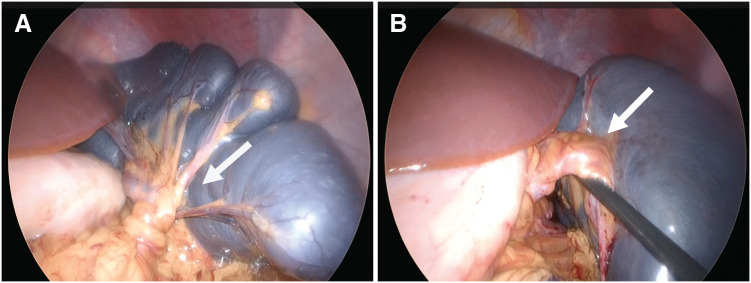
Intraoperative findings (**A**) Dark and congested spleen and complete 720° clockwise torsion of the spleen at the hilar pedicle (white arrow). (**B**) The pedicle after detorsion (white arrow). The peripheral ligaments of the spleen were absent.

The spleen was not fixed to the retroperitoneum and was markedly enlarged with dark purple discoloration, suggesting ischemia. The spleen was stem-twisted around the arteriovenous axis and returned to normal after rotating by 720° (**Fig. 2A**, **2B**). The color tone of the spleen gradually recovered with the release of torsion. Therefore, splenectomy was not performed, and the spleen was preserved. We considered it difficult to perform the splenectomy laparoscopically at that time due to the marked splenomegaly, so we ended the surgery with the plan to perform a two-stage splenectomy after the splenomegaly had improved.

**Fig. 3:**
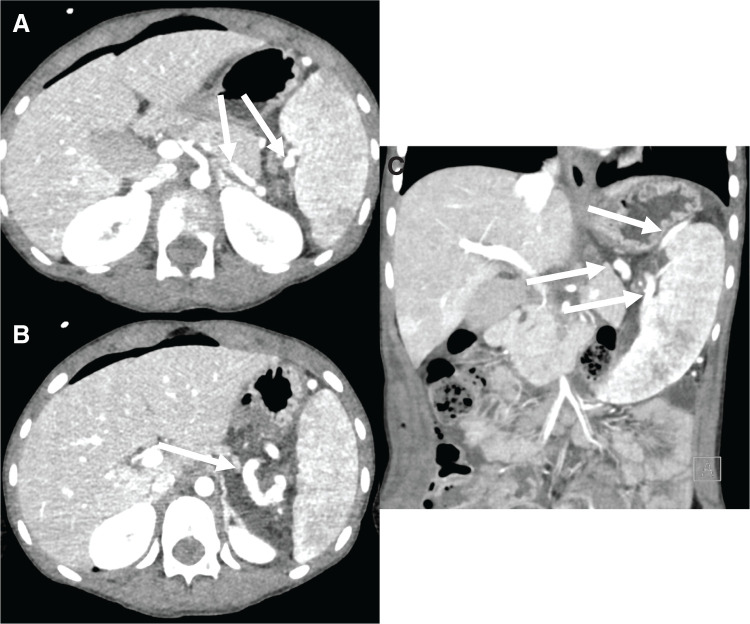
Abdominal CT scans taken on the day following detorsion (**A**) The blood flow of the main splenic vein was restored (white arrow). The axial (**B**) and coronal (**C**) images reveal enhanced spleen and collateral circulation from gastroepiplonic vein (white arrow).

On the 1st POD, the patient developed bleeding from the insertion site of the epidural anesthetic catheter, decreased blood pressure, worsening level of consciousness, and muscle weakness in both lower limbs. Blood tests showed a rapid fall in plasma platelet levels from 210000/μL before surgery to 7000/μL. Contrast-enhanced CT revealed restoration of the contrast effect in the spleen and resumed blood flow in the splenic arteriovenous system. The blood flow of the main splenic vein was also restored (**Fig. 3A**), but a large collateral hemorrhage channel was observed extending from the splenic hilum to the gastric vein (**Fig. 3B**, **3C**). He was diagnosed with splenic hyperfunction and thrombocytopenia due to resumption of blood flow to the spleen, resulting in epidural hemorrhage and hemorrhagic shock. Systemic management in the ICU and transfusion of platelets and red blood cells were initiated.

**Fig. 4:**
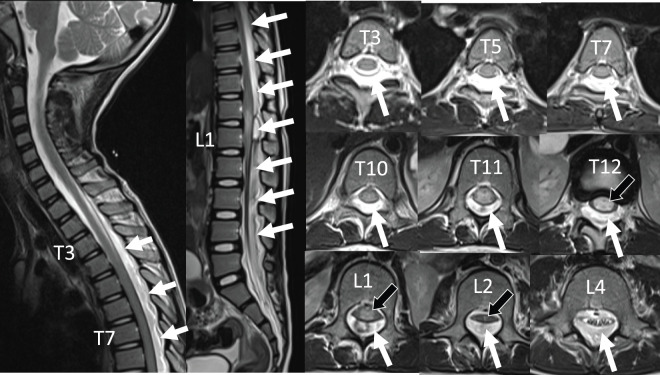
MRI, sagittal view and axil view in the T2 weighted images on 1 day after splenic detorsion. Spinal epidural hematoma was observed from T3 to L4 (white arrow) with spinal cord compression centered at T12-L1 (black arrow).

The patient continued to experience progressive muscle weakness in both the lower limbs, which led to complete paralysis. MRI revealed epidural hematoma in the thoracolumbar spine (**Fig. 4**), and a diagnosis of spinal cord injury caused by the epidural hematoma was made. Orthopedic surgeons performed hematoma removal and laminectomy on 1 day after splenic detorsion. The initial spine surgery was performed by hematoma evacuation and wash-out with minimized laminectomy level (T11-L2) to prevent growth disorders or kyphotic deformity due to multiple laminectomies, resulting in slight improvement in lower limb movement. Two drain tubes were placed, however, the occlusion in both drains was observed 8 hours postoperatively, and worsening of motor paralysis was noted. Emergency MRI revealed spinal cord compression due to recurrent hematoma (**Supplementary Fig. 1**). Emergent additional laminectomy (T5-9) and hematoma removal were done on the 2nd POD after splenic detorsion.

**Fig. 5:**
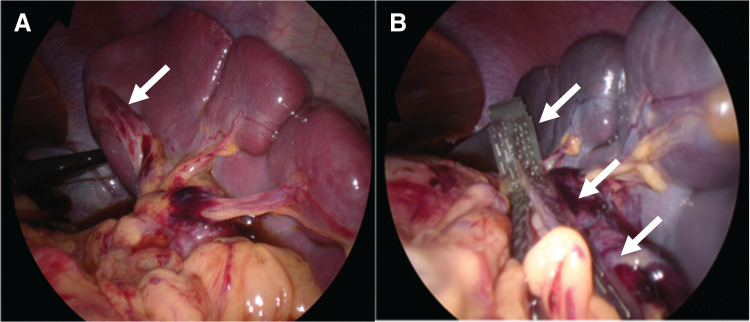
Second operative findings (**A**) The spleen color is restored and collateral circulation from the gastroepiploic vein is confirmed (white arrow). (**B**) The spleen pedicle including the collateral circulation was separated *en bloc* using an Electric Linear Stapler (white arrow).

He was unable to maintain platelets without dozens of units of platelet transfusion every day, and his general condition deteriorated, including the appearance of pleural effusion due to massive transfusion. Therefore, laparoscopic splenectomy was performed 5 days after detorsion. The color tone of the spleen improved to bright red, the splenomegaly improved, and an angry collateral hemorrhage around the splenic portal was observed (**Fig. 5A**). The splenic vessels were dissected *en bloc* using a stapler, and the spleen was removed (**Fig. 5B**). Pathological examination of the excised spleen showed overall hemorrhage and congestion, but no necrosis.

From the 1st day after splenectomy, the plasma platelet count rose to 200000/μL, and thereafter, blood transfusions were no longer required. His general condition stabilized and he was transferred to the general ward on the 6th POD. To improve his neurological condition, he underwent hyperbaric oxygen therapy and rehabilitation, which gradually improved his lower limb muscle strength. He was transferred to another hospital 47 days after splenectomy and was discharged after continuing rehabilitation for a further 2 months. Although he still had symptoms of bladder and rectal dysfunction, his neurological symptoms improved, and he was able to walk independently with the aid of a brace.

## DISCUSSION

Wandering spleen is extremely rare and is reported to occur in 0.2%–0.3% of patients who undergo splenectomy.^1,2)^ If left untreated, it can develop into splenic torsion and cause acute abdominal pain owing to congestion caused by splenic vein obstruction or ischemia caused by arterial obstruction. This condition can also cause complications, such as small bowel infarction, necrosis, and acute pancreatitis, and can be fatal.^3)^ Therefore, prompt treatment is required after diagnosis. The causes of wandering spleen can be divided into congenital and acquired factors. The former is caused by malformation or defects in the mesentery (gastrosplenic mesentery, splenocolic mesentery) that anchors the spleen in place, whereas the latter is believed to be caused by weakening of the ligaments anchoring the spleen due to pregnancy, multiple births, and trauma.^4–7)^ In addition, this condition is more common in childhood due to congenital factors and in young women due to acquired factors.^7,8)^

In this case, splenic infarction occurred 2 days after laparoscopic surgery for inguinal hernia, suggesting a causal relationship between the surgery and torsion of the splenic pedicle. However, as no manipulation of the upper abdomen, including the spleen, was performed during inguinal hernia surgery, and as there are no reports of similar splenic torsion caused by laparoscopic surgery, it is unlikely that the torsion of the splenic pedicle was caused by laparoscopic surgery alone. In this case, contrast CT performed after torsion release revealed a large collateral blood vessel extending from the spleen to the gastric venous system. The presence of this well-developed collateral blood vessel indicated a disturbance in the blood flow of the splenic vein for some time before the onset of splenic infarction. This patient had been actively engaged in gymnastics for several months before the event occurred. It is believed that these positional changes may have caused chronic splenic torsion and that the laparoscopic surgery created a space in the abdominal cavity, which may have led to further torsion and resulted in splenic infarction. It has been reported that 39% of patients diagnosed with splenic torsion have previously experienced similar abdominal pain^9)^; therefore, there can be cases of recurring chronic torsion.

The primary treatment for splenic torsion is splenectomy or splenopexy.^10)^ Since the risk of severe infection increases due to a decrease in immune function caused by splenectomy,^11)^ preservation of the spleen is recommended in cases in which blood flow can be restored by detorsion, and the spleen can be preserved.^12,13)^ Particularly, the incidence of severe infections and mortality after splenectomy is higher in children than in adults,^14)^ and the spleen should be preserved as much as possible.

In this case, we also chose to preserve the spleen because we observed the restoration of blood flow to the spleen and recovery of spleen color after detorsion. However, the patient developed a sudden decrease in platelet count due to hypersplenism and serious complications including epidural hemorrhage and spinal cord injury. Although there are scattered reports of cases in which splenic torsion has been associated with mild thrombocytopenia,^15,16)^ there have been no previous reports of marked thrombocytopenia developing after detorsion. In this case, blood flow was observed in the large collateral blood vessels after detorsion. The increased blood flow in the splenic artery due to abnormal perfusion via the splenic vein and large collateral blood vessels may have led to significant platelet destruction in the enlarged spleen.

We ultimately opted for splenectomy to address the prolonged thrombocytopenia. To improve his neurological prognosis, we needed to start rehabilitation and hyperbaric oxygen therapy as soon as possible. However, he required large amounts of platelet transfusions daily, and the resulting increase in pleural effusion and deterioration of his respiratory condition prevented us from starting treatment for his spinal cord injury. Partial splenectomy and continued conservative treatment were also options to preserve spleen function, but we opted for splenectomy as we believed it would be the most effective. Consequently, the patient no longer required blood transfusions from the day after the splenectomy, enabling us to promptly commence rehabilitation and hyperbaric oxygen therapy.

Although the mechanism is unknown, detorsion of the spleen can cause sudden thrombocytopenia and serious complications. When the spleen is preserved during surgery, measures to prepare for severe thrombocytopenia may be necessary, such as avoiding the placement of epidural catheters and drains as much as possible.

## CONCLUSIONS

We encountered a child who developed sudden and severe thrombocytopenia after splenic detorsion. When the spleen is preserved during surgery for torsion, preparation for thrombocytopenia is necessary.

## SUPPLEMENTARY MATERIALS

Supplementary Fig. 1 MRI, sagittal view and axil view in the T2 weighted images on 2 days after splenic detorsion. Recurrent spinal epidural hematoma was observed from T5 to L1 (white arrow) with spinal cord compression at T7, T12-L1. (black arrow).

## ACKNOWLEDGMENTS

We would like to thank Editage (www.editage.jp) for English language editing.

## DECLARATIONS

### Funding

None.

### Authors’ contributions 

Y In contributed to the data collection, postoperative care, and manuscript drafting.

YM performed surgeries on the spleen and contributed to manuscript review.

TY, KY, and JH performed spinal surgeries.

KO and RO contributed to the rehabilitation.

TA proofread this paper.

All other authors participated in the operation and postoperative management.

### Availability of data and materials

Deidentified patient data will be available upon reasonable request to the corresponding author.

### Ethics approval and consent to participate

Not applicable.

### Consent for publication

Informed consent was obtained from the patient and his parents for publication of the case report and accompanying images.

### Competing interests

The authors declare that they have no competing interests.
