# Supplementary figures and images for: Detorsion of Wandering Spleen Causes Significant Hypersplenism Resulting in Thrombocytopenia and Spinal Cord Injury: A Case Report
Source: Surg Case Rep. 2025 Aug 14;11(1):25-0111. doi: 10.70352/scrj.cr.25-0111 (PMC12377856; doi:10.70352/scrj.cr.25-0111)

Supplementary Fig.1

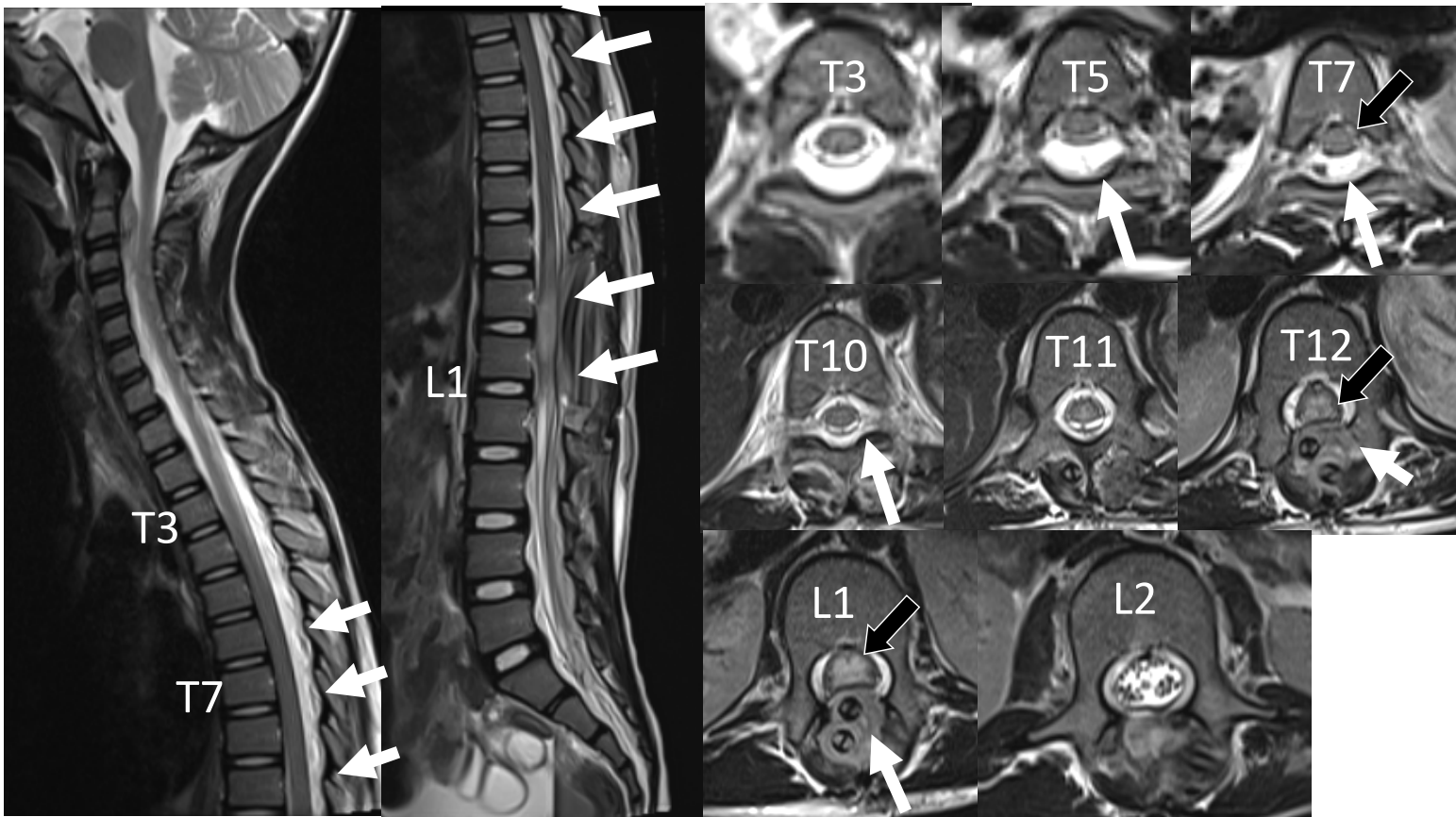

Supplement: Supplementary Fig. 1 [file scr-11-01-25-0111-s001.pdf]
